# From Bottles to Bruises: Partner Substance Use, Relationship Dynamics, and the Risk of Intimate Partner Violence in South Africa

**DOI:** 10.3390/ijerph23020160

**Published:** 2026-01-27

**Authors:** Judith Ifunanya Ani

**Affiliations:** Faculty of Medical and Health Sciences, Walter Sisulu University, Mthatha 5100, South Africa; jani@wsu.ac.za

**Keywords:** intimate partner violence, substance use, controlling behaviour, South Africa, gender-based violence

## Abstract

**Highlights:**

**Public health relevance—How does this work relate to a public health issue?**

- Intimate partner violence (IPV) and substance use are interlinked public health challenges that significantly increase women’s risk of physical, sexual, and emotional harm.
- This study examines the association between partner alcohol consumption and women’s experiences of IPV in South Africa, a setting with high levels of both harmful drinking and gender-based violence.

**Public health significance—Why is this work of significance to public health?**

- Findings demonstrate that women whose partners consume alcohol have substantially higher odds of experiencing all forms of IPV, reinforcing alcohol use as a critical but underaddressed driver of violence.
- The study provides population-level evidence that substance use operates as a structural risk factor for IPV, rather than merely an individual behavioural issue.

**Public health implications—What are the key implications or messages for practitioners, policy makers and/or researchers in public health?**

- IPV prevention and response strategies should integrate alcohol harm-reduction and substance-use interventions, particularly within primary healthcare and community-based programmes.
- Policymakers and researchers should strengthen cross-sectoral approaches linking public health, social services, and alcohol regulation to reduce IPV risk and improve women’s health outcomes.

**Abstract:**

Intimate partner violence (IPV) remains a persistent public health and human rights challenge globally, with South Africa experiencing some of the highest rates. This study investigates the intersection between partner substance use, controlling behaviours, and women’s risk of experiencing emotional, physical, and sexual IPV. Using nationally representative data from the South African Demographic and Health Survey (SADHS), a weighted sample of 2354 women was analysed. Findings show that 41.8% of women reported that their partners used alcohol and/or drugs, and IPV prevalence among this group was 36.38%. Multivariate logistic regression revealed that partner substance use more than doubled the risk of emotional violence and nearly tripled the risk of physical and sexual violence. Controlling behaviours also emerged as significant predictor, with affected women facing up to nine times higher odds of IPV. These findings highlight the urgent need for integrated intervention strategies that address substance abuse and coercive control within intimate relationships. Prevention efforts must be context-specific, targeting underlying behavioural and gendered power dynamics to reduce IPV and improve women’s safety.

## 1. Introduction

Intimate partner violence (IPV) remains one of the most pressing public health and human rights challenges globally. Defined as any behaviour within an intimate relationship that causes physical, emotional, or sexual harm to those in the relationship, IPV disproportionately affects women and is often perpetuated in contexts of gender inequality, poverty, and social marginalisation. According to the most recent global estimates by the World Health Organization (WHO) [1], approximately 30.4% of women aged 15 years and older worldwide—representing about 840 million women—have experienced physical and/or sexual violence by an intimate partner or sexual violence by a non-partner at least once in their lifetime, with little evidence of meaningful global decline over the past two decades. In South Africa, IPV constitutes a particularly severe social and public health concern, with recent national statistics indicating that approximately one in three women has experienced some form of IPV in her lifetime [2,3].

A substantial body of evidence identifies partner substance use—particularly alcohol and illicit drug use—as a key driver of IPV. Substance use impairs judgement, reduces inhibition, and heightens aggressive tendencies, thereby increasing the likelihood of violent behaviour within intimate relationships [4,5]. Studies consistently show that women whose partners abuse substances face significantly higher risks of emotional, physical, and sexual violence, with alcohol use most strongly associated with physical IPV [6,7,8]. Beyond increasing frequency, substance use has also been linked to greater severity, chronicity, and recurrence of IPV episodes. In South Africa, the normalisation of alcohol consumption in certain communities, combined with high unemployment and social stress, has contributed to elevated rates of both substance abuse and IPV [9,10,11].

Equally central to IPV dynamics is the role of controlling behaviour, often conceptualised as coercive control. Controlling behaviour refers to a pattern of domination that includes surveillance, restriction of movement, economic abuse, isolation from social networks, and emotional manipulation. Rather than isolated acts, these behaviours function as structural mechanisms through which power is exercised and violence is sustained. Such behaviours not only exacerbate the risk of IPV but frequently precede and facilitate physical and sexual abuse [12,13]. Women in relationships characterised by both controlling behaviour and substance abuse are therefore exposed to compounded vulnerability, particularly in patriarchal contexts where reporting is discouraged and access to justice or health services is limited [14].

Importantly, substance use and controlling behaviours often co-occur, reinforcing one another in ways that intensify IPV risk. Intoxication may amplify controlling tendencies by lowering self-regulation and accountability, while coercive control may normalise aggression and justify violent behaviour during substance use episodes. This interaction aligns with syndemic frameworks, which emphasise how multiple, coexisting risk factors interact synergistically to worsen health and social outcomes. Yet, despite this conceptual relevance, empirical studies rarely examine substance use and controlling behaviour together within integrated analytical models, particularly in low- and middle-income settings.

Scholars noted that substance use impairs cognitive functioning, lowers inhibitions, and increases the likelihood of aggressive behaviour, particularly in male-dominated relationships [15,16]. Alcohol, being the most socially accepted and widely consumed substance in many parts of sub-Saharan Africa, has been particularly implicated in IPV dynamics [17]. Globally, studies suggest that men who drink heavily are significantly more likely to perpetrate physical and sexual violence against their partners [1]. Studies demonstrate that alcohol-related aggression is a leading driver of domestic violence fatalities, while drug use, especially stimulants like methamphetamine, has been linked to coercive control and increased severity of IPV [18]. In South Africa, alcohol consumption is both culturally normalised and structurally embedded in male identity, particularly in informal settlements and post-apartheid urban townships, where recreational drinking often coexists with high unemployment, poverty, and community-level violence [19,20].

Relationship-level characteristics further shape IPV risk and its interaction with partner behaviour. Factors such as controlling behaviour, relationship duration, age disparities, and partner educational attainment have been shown to influence women’s exposure to violence. Younger women and those in newer relationships often face heightened vulnerability, especially when male partners are older, less educated, or economically dominant [21]. While educational attainment has been identified as a protective factor in some contexts, evidence suggests that in settings marked by entrenched gender inequality, higher education alone may not shield women from IPV—particularly where male partners perceive challenges to traditional gender hierarchies [17].

Despite extensive literature on IPV, notable gaps remain in the South African context. Much of the existing evidence relies on localised samples or examines substance use, controlling behaviour, or relationship characteristics in isolation. Few studies have employed nationally representative data to examine how these factors interact concurrently to shape women’s risk of emotional, physical, and sexual IPV. Given the widespread yet underreported nature of IPV in South Africa, such integrated analyses are critical for informing effective policy and programmatic responses.

This study addresses these gaps by analysing data from the 2016 South Africa Demographic and Health Survey (SADHS), ref. [22], to examine how partner substance use (alcohol and/or drugs), controlling behaviour, and relationship characteristics collectively influence women’s exposure to emotional, physical, and sexual IPV. By adopting an integrated, nationally representative approach, the study provides context-specific evidence that can guide targeted prevention strategies and interventions aimed at reducing IPV and improving women’s safety in South Africa.

## 2. Methods

### 2.1. Study Design

This study employed a cross-sectional analytical design using secondary data from the 2016 South Africa Demographic and Health Survey (SADHS). The SADHS is a nationally representative household survey conducted by Statistics South Africa in collaboration with the National Department of Health, Statistics South Africa (Stats SA), South African Medical Research Council (SAMRC), and international partners. It collects detailed information on demographic characteristics, health indicators, reproductive health, domestic violence, substance use behaviours among adult populations, and so on. The dataset used for this study specifically focuses on responses from women aged 18–49 who completed the domestic violence module, which includes questions on intimate partner violence, partner behaviours, and socio-demographic characteristics.

The collected range of sociodemographic and relationship-level variables include respondent age (18–49 years), educational attainment, marital status, number of children, and relationship duration. Partner characteristics included partner’s age and partner’s educational attainment. These variables were selected based on prior evidence linking sociodemographic and relationship factors to IPV risk and were included to control for potential confounding effects. Controlling behaviours was also measured using a set of items capturing coercive and restrictive partner behaviours. For example, respondents were asked whether their partner exhibited behaviours such as jealousy when the respondent spoke to other men, accusations of unfaithfulness, restriction of contact with family or friends, insistence on knowing the respondent’s whereabouts at all times, or limiting access to money. An example item is: “Does your partner try to limit your contact with your family or friends?” Responses were coded dichotomously. A composite variable was created to indicate the presence of any controlling behaviour, with respondents coded as “yes” if they reported at least one controlling behaviour.

### 2.2. Sample and Participants

The study was conducted among 2354 women aged 18–49 years currently or previously in an intimate relationship (ever-partnered women), and who responded to the domestic violence module of the South Africa Demographic and Health Survey (SADHS). Descriptive characteristics of the study sample are presented in the Section 3.

Respondents’ educational attainment ranged from no formal education to more than secondary education. Parity varied, with some women reporting no children and others reporting between one and five or more children. Relationship duration also differed substantially, ranging from less than one year to over 30 years. Information on partner characteristics was elicited including age, educational attainment and controlling behaviour.

### 2.3. Inclusion Criteria

The study included women aged 18–49 years who:

- Were currently or previously in an intimate relationship.
- Responded to the domestic violence module in the SADHS.
- Provided information on their partner’s alcohol and drug use.
- Provided complete responses to questions on IPV (emotional, physical, and sexual violence).

### 2.4. Measures

#### 2.4.1. Dependent (Outcome) Variables

The outcome variable was intimate partner violence (IPV). In the South Africa Demographic and Health Survey (SADHS), the domestic violence module was administered to ever-partnered women aged 18 years and older. IPV was measured as lifetime exposure, referring to violence experienced at any point since the age of 18. Violence perpetrated by a current partner (among currently partnered women) or the most recent partner (among formerly partnered women) was assessed by asking all ever-partnered women whether their partner had ever engaged in specific acts of emotional, physical, or sexual violence.

In this study, IPV was assessed using the standardised domestic violence module of the SADHS. The module captures three forms of IPV—emotional, physical, and sexual violence—experienced by women from a current or former intimate partner. Emotional violence was measured using three items, including whether the partner had ever insulted or humiliated the respondent, threatened her with harm, or made her feel bad about herself. Physical violence was assessed using seven items, covering acts such as slapping, pushing, hitting with a fist or object, kicking, choking, or threatening with a weapon. Sexual violence was measured using three items, including whether the respondent had ever been physically forced to have sexual intercourse or perform sexual acts against her will. A representative example item is: “Has your (current or most recent) partner ever hit, slapped, kicked, or done anything else to physically hurt you?” Responses were coded dichotomously (yes/no). For this study, each IPV type was analysed separately, and a composite binary variable (“any IPV”) was constructed to indicate whether a respondent experienced at least one form of emotional, physical, or sexual violence.

#### 2.4.2. Independent (Explanatory) Variable

The independent variable was partner substance use. Partner substance use was assessed through self-reported items asked of respondents about their partners’ behaviours. Two items were used: one assessing whether the partner consumed alcohol and another assessing whether the partner used drugs or other intoxicating substances. An item is: “Does your partner drink alcohol?” A similar item asked whether the partner used drugs. Responses were coded as yes/no. For analytical purposes, a binary composite variable was created to indicate any partner substance use, coded as 1 if the partner used alcohol and/or drugs, and 0 otherwise.

#### 2.4.3. Covariates

Sociodemographic and relationship-level covariates included respondent age (18–24, 25–34, 35–44, and 45+ years), educational attainment (no education, primary, secondary, and more than secondary), marital status (currently in a relationship or previously in a relationship), number of children (none, 1–4, and 5 or more), and relationship duration (categorised into years). Partner characteristics included partner’s age (18–24, 25–34, 35–44, and 45+ years) and partner’s educational attainment (no education, primary, secondary, and more than secondary). These variables were included to adjust for potential confounding in the association between partner substance use, controlling behaviour, and intimate partner violence.

### 2.5. Analysis

The analysis was conducted using Statistical Package for Social Sciences (SPSS) version 28 in three phases. Descriptive statistics were used to summarise the characteristics of the study sample and to determine the prevalence of IPV and partner substance use. Frequencies and percentages were calculated to provide nationally representative estimates. Bivariate analyses were conducted using Pearson’s chi-square tests to assess the association between IPV outcomes and key independent variables. These comparisons helped identify significant variations and population-level patterns in the risk of IPV. Finally, Multivariate analysis was carried out. Binary logistic regression models were estimated to examine the association between partner characteristics, relationship factors, and women’s experience of intimate partner violence (IPV). Four separate outcome variables were modelled—any IPV, emotional violence, physical violence, and sexual violence—each coded dichotomously (yes/no). The independent variables included in all regression models were respondent age, respondent educational attainment, number of children, relationship duration, partner’s educational attainment, partner’s age, partner controlling behaviour, and partner substance use. These variables were selected a priori based on existing empirical evidence and theoretical relevance linking sociodemographic, relational, and behavioural factors to IPV risk. All models were estimated using adjusted odds ratios (AORs) with 95% confidence intervals (CIs). The reference categories (RC) for categorical variables are indicated in the regression table. Statistical significance was assessed using a *p*-value threshold of *p* < 0.05, with results meeting this criterion marked accordingly.

Model explanatory power and goodness of fit were assessed using pseudo R^2^ statistics and the log-likelihood values, which are appropriate for logistic regression models. The overall contribution of the models was evaluated by examining the joint significance and stability of key predictors across the IPV outcomes. The consistency of effect sizes for partner controlling behaviour and substance use across all models supports the robustness of the analytic strategy. All analyses accounted for the complex survey design of the SADHS, including sampling weights, to ensure nationally representative estimates.

### 2.6. Ethical Considerations

The SADHS 2016 adhered to rigorous ethical protocols approved by the ICF Macro Institutional Review Board and the South African Department of Health’s Research Ethics Committee. Ethical clearance included protections for participants’ confidentiality, safety, and informed consent, particularly in administering the domestic violence module. For this secondary data analysis, no additional ethical approval was required. However, access to the dataset was granted through official DHS request procedures. The dataset used for this analysis and ethical protocols can be accessed at: http://goo.gl/ny8T6X (accessed on 31 August 2024).

## 3. Results

### 3.1. Prevalence of Intimate Partner Violence and Partner Substance Use

The analysis draws on data from the 2016 South Africa Demographic and Health Survey (SADHS), comprising a sample of 2354 women aged 18–49 years who responded to the domestic violence module. The findings reveal that IPV remains a critical concern in South Africa, with a significant proportion of women reporting experiences of abuse in their intimate relationships.

As shown in Table 1, specifically, 19.16% of the women reported experiencing emotional violence from their partners. This form of abuse, which includes behaviours such as verbal insults, threats, humiliation, or psychological manipulation, was the most commonly reported type of IPV. Physical violence—including acts such as hitting, slapping, pushing, or choking—was reported by 15.12% of respondents. Meanwhile, sexual violence, though less frequently reported, was still present, affecting 3.82% of the women surveyed. When these categories were combined, the overall prevalence of IPV among the sample was 24.60%, indicating that nearly one in four women had experienced at least one form of abuse from an intimate partner.

In addition to violence, the survey collected data on partner substance use, a factor increasingly recognised for its role in exacerbating IPV risk. Among the respondents, 40.91% reported that their partners consumed alcohol, while 3.14% reported that their partners used other drugs, such as marijuana or harder substances. When both alcohol and drug use were considered together, the combined prevalence of partner substance use rose to 41.80%. This figure suggests that more than two in five women in the sample were in relationships where their partners engaged in some form of substance use.

Taken together, these statistics highlight the high burden of IPV and the widespread presence of substance use among male partners in South Africa.

### 3.2. Partner Substance Use and Sociodemographic Characteristics

Table 2 presents the distribution of partner substance use across key sociodemographic and relationship characteristics. Overall, partner substance use was most commonly reported among women aged 25–34 years (39.74%) and 35–44 years (34.55%), together accounting for nearly three-quarters of all reported cases. A similar age pattern was observed among substance-using partners, with the largest proportion falling within the 35–44 years age group (39.06%).

In terms of educational attainment, the majority of women whose partners reported substance use had secondary education (69.92%), while a comparable proportion of their partners also had secondary education (68.97%). This suggests that partner substance use is most concentrated among couples with mid-level educational attainment rather than at the extremes of educational disadvantage or advantage.

Relationship and family characteristics further highlight important patterns. Most women reporting partner substance use had between one and four children (83.84%), and the majority were currently in a relationship (82.22%). Notably, over one-quarter of these relationships had lasted 0–4 years (27.64%), indicating that substance use exposure is particularly common in relatively early relationship stages.

Behavioural dynamics revealed a strong overlap between partner substance use and controlling behaviour. Nearly 46% (45.93%) of women whose partners used substances also reported that their partners exhibited controlling behaviours, a statistically significant association (*p* < 0.001).

Partner substance use was also strongly associated with all forms of intimate partner violence. Among women whose partners consumed alcohol and/or drugs, 28.15% reported emotional violence, compared to 19.16% in the overall sample. Similarly, 24.39% experienced physical violence, substantially higher than the sample prevalence of 15.12%, while 6.30% reported sexual violence—almost double the overall prevalence of 3.82%. When considering any form of IPV, 36.38% of women with substance-using partners reported at least one IPV experience, compared to 24.60% in the full sample (*p* < 0.001).

Taken together, these findings indicate that partner substance use is not only patterned by age, education, and relationship characteristics, but is also closely linked to controlling behaviours and markedly higher IPV prevalence.

### 3.3. Emotional Violence, Partner Substance Use and Associated Factors

Table 3 presents the distribution of emotional violence across selected sociodemographic, relationship, and partner-related characteristics. Emotional violence was most frequently reported among women aged 25–34 years (34.59%) and 35–44 years (37.03%), although age differences were not statistically significant (*p* = 0.19).

Educational attainment showed a clearer pattern. Women with secondary education accounted for the largest proportion of emotional violence cases (72.95%), followed by those with primary education (14.63%), while women with more than secondary education reported the lowest prevalence (9.53%). This association was statistically significant (*p* = 0.02), suggesting a potential protective effect of higher educational attainment.

With respect to family and relationship characteristics, emotional violence was most commonly reported among women with one to four children (82.04%), although parity differences were not statistically significant (*p* = 0.44). Marital status, however, was significantly associated with emotional violence (*p* < 0.001), with a higher proportion of emotional abuse reported among women who were currently in a relationship (76.05%) compared to those previously partnered (23.95%). Relationship duration showed a declining pattern, with higher prevalence observed in shorter relationships, though this association did not reach statistical significance (*p* = 0.11).

Partner characteristics revealed that emotional violence was most prevalent among women whose partners were aged 35–44 years (36.73%) and had secondary education (68.71%), although these associations were not statistically significant.

In contrast, partner behavioural factors demonstrated the strongest associations with emotional violence. A substantial majority of women whose partners exhibited controlling behaviour reported emotional violence (71.84%), compared to 28.16% among those whose partners did not, a difference that was highly significant (*p* < 0.001). Similarly, partner substance use was strongly associated with emotional violence. Emotional abuse was reported by 59.20% of women whose partners consumed alcohol, 8.20% of those whose partners used drugs, and 61.42% of those whose partners used alcohol and/or drugs (*p* < 0.001).

Overall, these findings highlight that while sociodemographic and relationship factors show mixed associations, partner controlling behaviour and substance use emerge as the most salient drivers of emotional violence.

### 3.4. Physical Violence, Partner Substance Use and Associated Factors

Table 4 presents the distribution of physical violence across sociodemographic, relationship, and partner-related characteristics. Physical violence varied significantly by age, educational attainment, marital status, partner characteristics, and partner substance use.

Age was significantly associated with physical violence (*p* = 0.01), with the highest prevalence reported among women aged 25–34 years (39.61%) and 35–44 years (32.87%). In contrast, women aged 45 years and older reported the lowest prevalence (15.17%), suggesting greater vulnerability or reporting of physical violence among younger and middle-aged women.

Educational attainment also demonstrated a strong association with physical violence (*p* < 0.001). Women with secondary education accounted for the majority of reported cases (72.19%), followed by those with primary education (17.70%), while women with more than secondary education reported substantially lower prevalence (7.02%). A similar pattern was observed for partner education, with higher prevalence among women whose partners had secondary (68.12%) or primary education (18.48%), and the lowest prevalence among those whose partners had education beyond secondary level (*p* < 0.001).

Marital status was significantly associated with physical violence (*p* = 0.01). Women who were previously in a relationship reported a higher prevalence (18.82%) compared to those currently partnered (81.18%), suggesting that experiences of physical violence may contribute to relationship dissolution.

Relationship duration and number of children were not significantly associated with physical violence. Nevertheless, higher prevalence was observed in shorter relationships (0–4 years; 26.97%), with a gradual decline as relationship duration increased, reaching the lowest levels among relationships lasting 30 years or more (2.25%). Physical violence was most commonly reported among women with one to four children (81.18%), reflecting their predominance in the sample.

Partner age also showed a statistically significant association with physical violence (*p* < 0.001). The highest prevalence was reported among women whose partners were aged 25–34 years (31.83%) and 35–44 years (35.29%), with lower prevalence among partners aged 45 years and older (27.68%).

Partner behavioural factors emerged as the strongest correlates of physical violence. A substantial majority of women whose partners exhibited controlling behaviour reported experiencing physical violence (75.28%), compared to 24.72% among those whose partners did not (*p* < 0.001). Partner substance use was similarly influential. Among women whose partners consumed alcohol, 64.33% experienced physical violence, compared to 35.67% among those whose partners did not (*p* < 0.001). When considering any substance use (alcohol and/or drugs), 67.42% of women with substance-using partners reported physical violence, compared to 32.58% among women whose partners did not use substances (*p* < 0.001).

Summarily, these findings indicate that while sociodemographic and relationship factors contribute to patterns of physical violence, partner controlling behaviour and substance use represent the most salient and consistent risk factors.

### 3.5. Sexual Violence, Partner Substance Use and Associated Factors

Table 5 presents the distribution of sexual violence across sociodemographic, relationship, and partner-related characteristics. Although the overall prevalence of sexual violence was lower than that of emotional and physical violence, clear patterns emerged in relation to partner behaviours and selected sociodemographic factors.

Age was not significantly associated with sexual violence (*p* = 0.70). Nevertheless, the largest proportions of reported cases occurred among women aged 25–34 years (35.56%) and 35–44 years (34.44%), indicating that sexual violence affects women across a broad age range without marked variation by age group.

Educational attainment was significantly associated with sexual violence (*p* = 0.04). Women with secondary education accounted for the majority of reported cases (78.89%), followed by those with primary education (14.44%), while women with more than secondary education reported substantially lower prevalence (3.33%), suggesting a potential protective role of higher educational attainment. A similar pattern was observed for partner education, with the highest prevalence reported among women whose partners had secondary education (75.86%) and the lowest among those whose partners had education beyond secondary level (*p* = 0.03).

Parity showed a marginal association with sexual violence (*p* = 0.08). Sexual violence was most frequently reported among women with one to four children (78.89%), followed by those with five or more children (15.56%), while women with no children reported the lowest prevalence (5.56%). Marital status was significantly associated with sexual violence (*p* < 0.001), with a higher proportion of cases reported among women who were currently in a relationship (68.89%) compared to those previously partnered (31.11%), suggesting that sexual violence may persist within ongoing relationships.

Relationship duration and partner age were not significantly associated with sexual violence. However, most cases occurred among women in shorter to mid-term relationships, with prevalence declining in relationships lasting 25 years or more, a pattern that may reflect lower incidence or underreporting among older cohorts.

Partner behavioural factors demonstrated the strongest associations with sexual violence. A striking 82.22% of women whose partners exhibited controlling behaviours reported experiencing sexual violence, compared to 17.78% among those whose partners were not controlling (*p* < 0.001). Partner substance use was similarly influential. Sexual violence was reported by 68.89% of women whose partners consumed alcohol and by 10.00% of those whose partners used drugs. When considering any substance use (alcohol and/or drugs), 68.89% of women with substance-using partners reported sexual violence, compared to 31.11% among women whose partners did not use substances (*p* < 0.001).

These findings indicate that while sexual violence cuts across sociodemographic groups, partner controlling behaviour and substance use emerge as the most salient and consistent correlates, underscoring the central role of partner behaviour in shaping women’s risk of sexual violence.

### 3.6. Intimate Partner Violence, Partner Substance Use and Associated Factors

Table 6 presents the prevalence of overall intimate partner violence (IPV), defined as experiencing at least one form of emotional, physical, or sexual violence, across sociodemographic, relationship, and partner-related characteristics. Overall IPV was significantly associated with educational attainment, marital status, partner behaviour, and partner substance use, while age and relationship duration showed weaker or non-significant associations.

Age was not significantly associated with overall IPV (*p* = 0.10). Nevertheless, the highest prevalence was observed among women aged 25–34 years (37.65%) and 35–44 years (34.72%), while lower prevalence was reported among women aged 18–24 years (10.54%) and 45 years and older (17.10%), suggesting variation in exposure or reporting across the life course.

Educational attainment demonstrated a strong and statistically significant association with IPV (*p* < 0.001). Women with secondary education accounted for the majority of IPV cases (71.50%), followed by those with primary education (15.72%), whereas women with more than secondary education reported substantially lower prevalence (9.50%), indicating a potential protective role of higher education. A similar pattern was observed for partner education, with IPV most prevalent among women whose partners had secondary (68.82%) or primary education (15.94%), and lowest among those whose partners had education beyond secondary level (*p* = 0.04).

Marital status was significantly associated with IPV (*p* < 0.001). A higher proportion of IPV was reported among women who were previously in a relationship (21.59%) compared to those currently partnered (78.41%), suggesting that experiences of violence may contribute to relationship dissolution or be more readily disclosed following separation.

Relationship duration and number of children were not significantly associated with IPV. However, higher prevalence was observed in shorter relationships, particularly those lasting 0–4 years (24.01%) and 5–9 years (22.28%), with prevalence declining as relationship duration increased, reaching the lowest levels among relationships lasting 25 years or more (<7%). IPV was most frequently reported among women with one to four children (81.69%), reflecting their predominance in the sample.

Partner age did not show a statistically significant association with IPV (*p* = 0.12), although most cases occurred among women whose partners were aged 25–34 years (29.07%) and 35–44 years (35.68%), indicating a concentration of IPV within these age groups.

Partner behavioural factors emerged as the most salient correlates of overall IPV. Women whose partners exhibited controlling behaviour were substantially more likely to experience IPV (68.91%) compared to those whose partners did not (31.09%), a highly significant association (*p* < 0.001). Partner substance use was similarly influential. IPV was reported by 59.76% of women whose partners consumed alcohol, compared to 40.24% among those whose partners did not (*p* < 0.001). Partner drug use was also significantly associated with IPV (*p* < 0.001), despite lower overall prevalence.

When alcohol and drug use were combined, 61.83% of women whose partners engaged in any form of substance use reported experiencing IPV, compared to 38.17% among women whose partners did not use substances (*p* < 0.001).

Taken together, these findings demonstrate that while sociodemographic and relationship characteristics contribute to IPV patterns, partner controlling behaviours and substance use represent the most consistent and powerful correlates of women’s overall IPV risk.

### 3.7. Logistic Regression on Predictors of IPV and Its Forms

Table 7 presents the results of the logistic regression analyses examining factors associated with women’s experience of overall IPV and its specific forms (emotional, physical, and sexual violence). The regression models included respondent age, educational attainment, number of children, relationship duration, partner’s age, partner’s educational attainment, partner controlling behaviour, and partner substance use.

Women’s age was significantly associated with IPV outcomes. Compared to women aged 18–24 years, those aged 35–44 years and 45 years and older had significantly lower odds of experiencing overall IPV and emotional violence. Specifically, women aged 35–44 years had lower odds of reporting any IPV and emotional violence, while women aged 45 years and above also showed reduced odds for these outcomes.

Educational attainment was not a statistically significant predictor of overall IPV or its individual forms in the adjusted models. Similarly, the number of children was not significantly associated with overall IPV, emotional violence, or physical violence. However, women with five or more children had higher odds of experiencing sexual violence compared to women with no children, although this association did not reach statistical significance.

Relationship duration showed a significant association with emotional violence. Women in relationships lasting 10–14 years had significantly higher odds of experiencing emotional violence compared to those in relationships of 0–4 years. Relationship duration was not significantly associated with physical or sexual violence.

Partner-related characteristics showed mixed associations. Partner’s educational attainment was not significantly associated with IPV outcomes. Partner’s age was significantly associated with physical violence, with women whose partners were aged 35–44 years having lower odds of experiencing physical violence compared to those whose partners were aged 18–24 years.

Partner controlling behaviour emerged as a strong and consistent predictor across all IPV outcomes. Women whose partners exhibited controlling behaviours had substantially higher odds of experiencing overall IPV, emotional violence, physical violence, and sexual violence compared to women whose partners did not exhibit such behaviours.

Partner substance use was also significantly associated with IPV outcomes. Women whose partners used alcohol and/or drugs had higher odds of experiencing overall IPV, emotional violence, physical violence, and sexual violence compared to women whose partners did not report substance use.

## 4. Discussion

This study examined how partner substance use, controlling behaviour, and relationship characteristics interact to shape women’s experiences of intimate partner violence (IPV) in South Africa. Drawing on nationally representative data from the 2016 South Africa Demographic and Health Survey (SADHS), the findings contribute to the growing body of evidence on IPV in sub-Saharan Africa and speak directly to global public health and human rights frameworks that recognise violence against women as a major health and development concern.

The finding that nearly one in four women (24.6%) reported experiencing at least one form of IPV—emotional, physical, or sexual—aligns with previous national estimates [1,2]. Emotional violence emerged as the most prevalent form of abuse, followed by physical and sexual violence. This pattern reinforces global public health perspectives that emphasise IPV as a multifaceted phenomenon, in which psychological abuse is both widespread and deeply harmful, often co-occurring with or preceding physical forms of violence [4].

A consistent and compelling finding across all forms of IPV was the significant role of partner substance use. Women whose partners consumed alcohol or drugs were more than twice as likely to experience emotional, physical, or sexual violence compared to those whose partners did not. These findings are consistent with prior research demonstrating a strong association between substance use—particularly alcohol—and IPV perpetration [8,15,16]. From a global health standpoint, substance use is widely recognised as a key modifiable risk factor for violence, as alcohol-related disinhibition and drug-induced impulsivity reduce behavioural control and increase aggressive responses to conflict or perceived disrespect [18]. In the South African context, where alcohol consumption is socially normalised, particularly in informal settlements and among marginalised men [9,10], these risks are further intensified by poverty, unemployment, and chronic social stress.

Beyond substance use, partner controlling behaviour emerged as a particularly powerful predictor of IPV. Women whose partners exhibited controlling behaviours were over six times more likely to experience overall IPV, with even higher odds observed for emotional and sexual violence. This finding reinforces global evidence that coercive control is central to the perpetuation of IPV [4,12,13]. Behaviours such as isolating women from support networks, restricting movement, and exerting financial control create relational environments in which abuse is normalised, concealed, and sustained. Notably, the co-occurrence of controlling behaviour and substance use appears especially dangerous, as both factors interact to reinforce domination, escalation, and harm.

These findings align with global ecological and syndemic frameworks, which emphasise that IPV does not arise from a single cause but from the interaction of behavioural, relational, and structural factors [10]. Within this perspective, partner substance use and controlling behaviour operate at the relationship level but are embedded within broader systems of gender inequality and social disadvantage that shape women’s vulnerability to violence.

Demographic and relationship characteristics provided additional context for understanding IPV risk. Age appeared protective, with women aged 35–44 years and 45 years and older being less likely to experience IPV compared to younger women. This may reflect greater relationship stability or exit from abusive partnerships over time. Alternatively, it may indicate underreporting among older women due to stigma, normalisation of violence, or generational differences in perceptions of abuse [21]. Relationship duration showed mixed effects, with elevated risks of emotional violence observed in relationships lasting 10–14 years and 20–24 years, suggesting that emotional abuse may persist or escalate over time even in long-term partnerships.

The protective role of education was less clear in the multivariate analysis, with no significant associations observed between respondent or partner education and IPV risk. However, descriptive and bivariate findings indicated that women with more than secondary education experienced the lowest IPV prevalence. This pattern is consistent with studies suggesting that education may enhance autonomy and access to resources, although in highly patriarchal settings, education alone may not be sufficient to counter deeply entrenched power imbalances, particularly where male partners feel threatened or emasculated [17].

Another important finding was the elevated IPV risk among women who were previously in relationships. These women were significantly more likely to report IPV, particularly emotional and sexual violence, suggesting that violence may have contributed to relationship dissolution or that abuse may persist following separation. This highlights the importance of post-relationship protection and support mechanisms, especially for women attempting to exit violent partnerships.

Overall, this study affirms that partner substance use and controlling behaviour are not merely correlated with IPV but represent strong and independent predictors across all forms of violence examined. While demographic and relationship characteristics provide important background context, partner behaviour emerges as the most consistent driver of women’s lived experiences of IPV in South Africa. These findings support the syndemic model linking IPV, substance use, and structural inequalities [10], underscoring the need for integrated, multi-level prevention strategies.

In conclusion, this study contributes nationally representative evidence to the literature on IPV and highlights critical entry points for intervention. Addressing IPV in South Africa requires public health approaches that reduce harmful alcohol and drug use, challenge controlling and unequal gender norms, and respond to the broader social and economic conditions that sustain violence. Interventions must be tailored to women’s diverse relationship stages and community contexts to be effective and sustainable.

## 5. Conclusions and Recommendations

This study provides nationally representative evidence on the relationship between partner substance use, controlling behaviour, and intimate partner violence (IPV) among women in South Africa. More than one in four women reported experiencing at least one form of emotional, physical, or sexual IPV, underscoring the continued public health significance of partner violence in the country.

Across all forms of IPV, partner substance use and controlling behaviour emerged as the most consistent and robust correlates, outweighing the influence of sociodemographic and relationship characteristics. These findings highlight the central role of partner behaviour in shaping women’s vulnerability to violence and identify critical behavioural entry points for effective prevention.

From a policy and practice perspective, the findings reinforce the need to address IPV as a gendered public health issue rooted in power, control, and social norms, rather than viewing it solely as private or relational. Doing this will help in the pursuit of attainment of Sustainable Development Goal (SDG) 5 aimed at achieving gender equality and empowering women and girls [1]. Partner violence occurs within unequal gender relations and is reinforced by structural conditions that normalise male dominance, alcohol misuse, and women’s economic and social dependence. Policy responses must therefore move beyond crisis intervention to address these underlying drivers.

First, intervention programmes for perpetrators should explicitly integrate substance use treatment with violence prevention strategies. Batterer intervention programmes, probation services, and court-mandated counselling should include structured components that address alcohol and drug misuse alongside accountability for abusive behaviour. This may involve routine substance use screening in IPV cases, clear referral pathways to addiction treatment, and coordinated case management between substance use and violence prevention services.

Second, controlling behaviours should be formally recognised and addressed as a core component of IPV, rather than treated as secondary or non-violent. Early identification of coercive control—such as social isolation, financial restriction, and monitoring of women’s movements—should be incorporated into healthcare settings, social services, and community-based screening tools. Intervening at this stage may help prevent escalation to more severe forms of abuse.

Third, prevention efforts must directly engage with harmful masculinities and gender norms that legitimise control and violence against women. Community- and school-based programmes should prioritise men and boys, particularly in high-risk environments, to promote non-violent, gender-equitable relationship norms. Such initiatives should challenge the association between masculinity, alcohol use, and dominance, while promoting alternative models of masculinity grounded in respect, emotional regulation, and shared decision-making.

Fourth, survivor support should extend beyond immediate crisis response. Women exiting abusive relationships—especially those previously in relationships—require sustained access to safe housing, economic support, psychosocial care, and legal protection. Strengthening linkages between IPV services, social protection systems, and employment or skills-development programmes is essential to reducing women’s long-term vulnerability to violence.

Overall, this study reinforces the need for multi-level, gender-responsive approaches to IPV prevention in South Africa. Addressing partner substance use and controlling behaviour as root causes of IPV requires coordinated action across health, justice, social development, and community sectors. By situating IPV within broader systems of gender inequality, substance misuse, and structural disadvantage, policies and interventions can move beyond awareness-raising toward transforming the conditions that allow violence against women to persist.

## 6. Limitations of the Study

Despite its strengths, including the use of nationally representative data, this study has several limitations that should be acknowledged. First, the analysis is based on cross-sectional data from the 2016 South Africa Demographic and Health Survey, which limits the ability to establish causal relationships between partner substance use, controlling behaviour, and IPV. The observed associations therefore reflect correlations rather than temporal or causal pathways.

Second, IPV, partner substance use, and controlling behaviours were measured using self-reported responses, which may be subject to recall bias or social desirability bias. Given the sensitive nature of IPV, underreporting is likely, particularly among older women or those in long-term relationships, potentially leading to conservative prevalence estimates.

Third, the DHS measures capture violence perpetrated by a current or the most recent partner but do not fully account for the frequency, severity, or timing of abuse. As a result, the analysis cannot distinguish between isolated incidents and chronic or escalating patterns of violence. Similarly, substance use was measured broadly and does not capture intensity, frequency, or timing of alcohol or drug consumption relative to violent episodes.

Finally, although the study controlled for key sociodemographic, relationship, and partner characteristics, unmeasured confounding factors—such as mental health conditions, childhood exposure to violence, or community-level norms—may also influence IPV risk and could not be examined within the available data.

These limitations should be considered when interpreting the findings; however, they do not detract from the study’s contribution in highlighting robust and policy-relevant associations between partner substance use, controlling behaviour, and IPV at the population level.

## Figures and Tables

**Table 1:** Prevalence of Intimate Partner Violence and Partner Substance Use.

Indicator	n (%)
Sample Size	2354
Emotional Violence Prevalence	451 (19.16%)
Physical Violence Prevalence	356 (15.12%
Sexual Violence Prevalence	90 (3.82%)
Intimate Partner Violence Prevalence	579 (24.60%)
Partner’s Alcohol Consumption Prevalence	963 (40.91%)
Partner’s Drug Usage Prevalence	74 (3.14%)
Partner’s Substance Usage Prevalence	984 (41.80%)

**Table 2:** Partner Substance Use and Sociodemographic Characteristics.

Variable	Weighted (n)	Weighted (%)	Substance Use (%)	*p*-Value
Age Group				0.04
18–24	192	8.16	9.45	
25–34	890	37.81	39.74	
35–44	868	36.87	34.55	
45+	404	17.16	16.26	
Educational Attainment				0.42
No education	85	3.61	3.56	
Primary	293	12.45	13.72	
Secondary	1659	70.48	69.92	
More than secondary	317	13.47	12.80	
Number of children				0.42
None	178	7.56	7.93	
1–4	1962	83.35	83.84	
5+	214	9.09	8.23	
Marital Status				<0.001
Previously in a relationship	339	14.40	17.78	
Currently in a relationship	2015	85.60	82.22	
Relationship Duration				0.09
0–4 years	598	25.40	27.64	
5–9 years	548	23.28	23.78	
10–14 years	401	17.03	15.04	
15–19 years	361	15.34	16.26	
20–24 years	232	9.86	8.74	
25–29 years	161	6.84	6.40	
30+ years	53	2.25	2.13	
Partner’s Education				0.31
No education	117	5.99	5.26	
Primary	276	14.12	12.95	
Secondary	1303	66.68	68.97	
More than secondary	258	13.20	12.82	
Partner’s Age				<0.001
18–24	49	2.43	3.09	
25–34	537	26.65	30.66	
35–44	785	38.96	39.06	
45+	644	31.96	27.19	
Partner has controlling behaviours				<0.001
No	1548	65.76	54.07	
Yes	806	34.24	45.93	
Respondent experienced emotional violence				<0.001
No	1903	80.84	71.85	
Yes	451	19.16	28.15	
Respondent experienced physical violence				<0.001
No	1998	84.88	75.61	
Yes	356	15.12	24.39	
Respondent experienced sexual violence				<0.001
No	2264	96.18	93.70	
Yes	90	3.82	6.30	
Respondent experienced IPV				<0.001
No	1775	75.40	63.62	
Yes	579	24.60	36.38	

**Table 3:** Emotional Violence, Partner Substance Use and Associated Factors.

Variable	Weighted n	Weighted %	Emotional Violence (%)	*p*-Value
Age Group				0.19
18–24	192	8.16	10.20	
25–34	890	37.81	34.59	
35–44	868	36.87	37.03	
45+	404	17.16	18.18	
Educational Attainment				0.02
No education	85	3.61	2.88	
Primary	293	12.45	14.63	
Secondary	1659	70.48	72.95	
More than secondary	317	13.47	9.53	
Number of children				0.44
None	178	7.56	7.32	
1–4	1962	83.35	82.04	
5+	214	9.09	10.65	
Marital Status				<0.001
Previously in a relationship	339	14.40	23.95	
Currently in a relationship	2015	85.60	76.05	
Relationship Duration				0.11
0–4 years	598	25.40	21.51	
5–9 years	548	23.28	22.17	
10–14 years	401	17.03	20.84	
15–19 years	361	15.34	15.74	
20–24 years	232	9.86	10.20	
25–29 years	161	6.84	6.43	
30+ years	53	2.25	3.10	
Partner’s Education				0.35
No education	117	5.99	6.44	
Primary	276	14.12	14.72	
Secondary	1303	66.68	68.71	
More than secondary	258	13.20	10.12	
Partner’s Age				0.71
18–24	49	2.43	2.62	
25–34	537	26.65	26.24	
35–44	785	38.96	36.73	
45+	644	31.96	34.40	
Partner has controlling behaviours				<0.001
No	1548	65.76	28.16	
Yes	806	34.24	71.84	
Partner drinks alcohol				<0.001
No	1391	59.09	40.80	
Yes	963	40.91	59.20	
Partner takes drugs				<0.001
No	2280	96.86	91.80	
Yes	74	3.14	8.20	
Partner indulges in substance use				<0.001
No	1370	58.20	38.58	
Yes	984	41.80	61.42	

**Table 4:** Physical Violence, Partner Substance Use and Associated Factors.

Variable	Weighted n	Weighted (%)	Physical Violence (%)	*p*-Value
Age Group				0.01
18–24	192	8.16	12.36	
25–34	890	37.81	39.61	
35–44	868	36.87	32.87	
45+	404	17.16	15.17	
Educational Attainment				<0.001
No education	85	3.61	3.09	
Primary	293	12.45	17.70	
Secondary	1659	70.48	72.19	
More than secondary	317	13.47	7.02	
Number of children				0.39
None	178	7.56	7.87	
1–4	1962	83.35	81.18	
5+	214	9.09	10.96	
Marital Status				0.01
Previously in a relationship	339	14.40	18.82	
Currently in a relationship	2015	85.60	81.18	
Relationship Duration				0.49
0–4 years	598	25.40	26.97	
5–9 years	548	23.28	23.31	
10–14 years	401	17.03	19.94	
15–19 years	361	15.34	12.64	
20–24 years	232	9.86	8.43	
25–29 years	161	6.84	6.46	
30+ years	53	2.25	2.25	
Partner’s Education				<0.001
No education	117	5.99	6.16	
Primary	276	14.12	18.48	
Secondary	1303	66.68	68.12	
More than secondary	258	13.20	7.25	
Partner’s Age				<0.001
18–24	49	2.43	5.19	
25–34	537	26.65	31.83	
35–44	785	38.96	35.29	
45+	644	31.96	27.68	
Partner has controlling behaviours				<0.001
No	1548	65.76	24.72	
Yes	806	34.24	75.28	
Partner drinks alcohol				<0.001
No	1391	59.09	35.67	
Yes	963	40.91	64.33	
Partner takes drugs				<0.001
No	2280	96.86	90.17	
Yes	74	3.14	9.83	
Partner indulges in substance use				<0.001
No	1370	58.20	32.58	
Yes	984	41.80	67.42	

**Table 5:** Sexual Violence, Partner Substance Use and Associated Factors.

Variable	Weighted n	Weighted (%)	Sexual Violence (%)	*p*-Value
Age Group				0.70
18–24	192	8.16	11.11	
25–34	890	37.81	35.56	
35–44	868	36.87	34.44	
45+	404	17.16	18.89	
Educational Attainment				0.04
No education	85	3.61	3.33	
Primary	293	12.45	14.44	
Secondary	1659	70.48	78.89	
More than secondary	317	13.47	3.33	
Number of children				0.08
None	178	7.56	5.56	
1–4	1962	83.35	78.89	
5+	214	9.09	15.56	
Marital Status				<0.001
Previously in a relationship	339	14.40	31.11	
Currently in a relationship	2015	85.60	68.89	
Relationship Duration				0.58
0–4 years	598	25.40	23.33	
5–9 years	548	23.28	20.00	
10–14 years	401	17.03	20.00	
15–19 years	361	15.34	18.89	
20–24 years	232	9.86	8.89	
25–29 years	161	6.84	4.44	
30+ years	53	2.25	4.44	
Partner’s Education				0.03
No education	117	5.99	10.34	
Primary	276	14.12	12.07	
Secondary	1303	66.68	75.86	
More than secondary	258	13.20	1.72	
Partner’s Age				0.26
18–24	49	2.43	4.84	
25–34	537	26.65	33.87	
35–44	785	38.96	37.10	
45+	644	31.96	24.19	
Partner has controlling behaviours				<0.001
No	1548	65.76	17.78	
Yes	806	34.24	82.22	
Partner drinks alcohol				<0.001
No	1391	59.09	31.11	
Yes	963	40.91	68.89	
Partner takes drugs				<0.001
No	2280	96.86	90.00	
Yes	74	3.14	10.00	
Partner indulges in substance use				<0.001
No	1370	58.20	31.11	
Yes	984	41.80	68.89	

**Table 6:** Intimate Partner Violence, Partner Substance Use and Associated Factors.

Variable	Weighted n	Weighted (%)	IPV (%)	*p*-Value
Age Group				0.10
18–24	192	8.16	10.54	
25–34	890	37.81	37.65	
35–44	868	36.87	34.72	
45+	404	17.16	17.10	
Educational Attainment				<0.001
No education	85	3.61	3.28	
Primary	293	12.45	15.72	
Secondary	1659	70.48	71.50	
More than secondary	317	13.47	9.50	
Number of children				0.11
None	178	7.56	7.08	
1–4	1962	83.35	81.69	
5+	214	9.09	11.23	
Marital Status				<0.001
Previously in a relationship	339	14.40	21.59	
Currently in a relationship	2015	85.60	78.41	
Relationship Duration				0.35
0–4 years	598	25.40	24.01	
5–9 years	548	23.28	22.28	
10–14 years	401	17.03	19.86	
15–19 years	361	15.34	14.34	
20–24 years	232	9.86	9.67	
25–29 years	161	6.84	6.91	
30+ years	53	2.25	2.94	
Partner’s Education				0.04
No education	117	5.99	6.00	
Primary	276	14.12	15.94	
Secondary	1303	66.68	68.82	
More than secondary	258	13.20	9.24	
Partner’s Age				0.12
18–24	49	2.43	3.52	
25–34	537	26.65	29.07	
35–44	785	38.96	35.68	
45+	644	31.96	31.72	
Partner has controlling behaviours				<0.001
No	1548	65.76	31.09	
Yes	806	34.24	68.91	
Partner drinks alcohol				<0.001
No	1391	59.09	40.24	
Yes	963	40.91	59.76	
Partner takes drugs				<0.001
No	2280	96.86	92.40	
Yes	74	3.14	7.60	
Partner indulges in substance use				<0.001
No	1370	58.20	38.17	
Yes	984	41.80	61.83	

**Table 7:** Logistic Regression Results: Predictors of IPV and Its Forms.

Logistic Regression				
Variables	IPV	Emotional Violence	Physical Violence	Sexual Violence
Age				
18–24	RC	RC	RC	RC
25–34	0.68 [0.42–1.11]	0.54 [0.32–0.91] *	0.91 [0.52–1.58]	0.71 [0.28–1.80]
35–44	0.51 [0.29–0.91] *	0.44 [0.24–0.81] **	0.78 [0.41–1.49]	0.51 [0.16–1.65]
45+	0.49 [0.24–0.99] *	0.44 [0.21–0.94] *	0.82 [0.36–1.86]	1.19 [0.26–5.53]
Education				
No education	RC	RC	RC	RC
Primary	0.97 [0.46–2.04]	1.00 [0.43–2.31]	1.40 [0.59–3.33]	0.81 [0.19–3.44]
Secondary	0.77 [0.38–1.56]	1.00 [0.45–2.21]	0.91 [0.40–2.10]	0.70 [0.18–2.72]
More than secondary	0.64 [0.29–1.44]	0.74 [0.29–1.83]	0.66 [0.25–1.75]	0.15 [0.01–1.69]
Number of children				
None	RC	RC	RC	RC
1–4	0.88 [0.56–1.40]	0.76 [0.46–1.26]	0.80 [0.47–1.36]	1.33 [0.44–4.02]
5+	1.32 [0.71–2.47]	1.01 [0.51–1.99]	1.17 [0.57–2.39]	3.12 [0.75–12.91]
Relationship Duration				
0–4 years	RC	RC	RC	RC
5–9 years	1.32 [0.93–1.88]	1.52 [1.02–2.27] *	1.41 [0.94–2.12]	1.02 [0.48–2.18]
10–14 years	1.74 [1.16–2.59] *	1.84 [1.18–2.88] **	1.62 [1.01–2.57] *	1.01 [0.42–2.45]
15–19 years	1.24 [0.79–1.95]	1.56 [0.96–2.57]	1.02 [0.59–1.75]	1.13 [0.44–2.93]
20–24 years	1.67 [0.97–2.88]	1.98 [1.09–3.57] *	1.12 [0.57–2.21]	0.15 [0.02–1.38]
25–29 years	1.29 [0.66–2.52]	0.88 [0.40–1.91]	1.41 [0.65–3.07]	0.38 [0.06–2.26]
30+ years	2.09 [0.73–5.97]	2.13 [0.69–1.91]	0.94 [0.25–3.54]	0.36 [0.03–4.11]
Partner’s Education				
No education	RC	RC	RC	RC
Primary	1.06 [0.59–1.90]	0.86 [0.45–1.63]	1.07 [0.55–2.12]	0.33 [0.10–1.12]
Secondary	1.10 [0.64–1.89]	0.97 [0.54–1.74]	0.88 [0.47–1.65]	0.52 [0.19–1.45]
More than secondary	0.92 [0.47–1.81]	0.97 [0.47–20.01]	0.66 [0.29–1.49]	0.13 [0.01–1.24]
Partner’s Age				
18–24	RC	RC	RC	RC
25–34	0.79 [0.37–1.68]	1.22 [0.51–2.92]	0.46 [0.21–1.01] *	0.83 [0.21–3.26]
35–44	0.79 [0.36–1.75]	1.55 [0.62–3.87]	0.39 [0.17–0.91] *	0.79 [0.18–3.44]
45+	1.11 [0.48–2.57]	2.36 [0.90–6.18]	0.45 [0.18–1.10]	0.77 [0.15–3.95]
Partner’s Controlling Behaviour				
No	RC	RC	RC	RC
Yes	6.56 [5.14–8.37] **	7.10 [5.39–9.36] **	6.57 [4.88–8.84] **	8.91 [4.38–18.13] **
Substance Use				
No	RC	RC	RC	RC
Yes	2.48 [1.95–3.17] **	2.26 [1.73–2.96] **	2.72 [2.04–3.64] **	2.83 [1.54–5.21] **
Cons	0.18 [0.05–0.42] **	0.06 [0.02–0.20] **	0.12 [0.04–0.40] **	0.02 [0.00–0.17] **

Notes: RC = Reference category; Values are adjusted odds ratios (ORs) with 95% confidence intervals (CIs) shown in brackets; * = *p* < 0.05; ** = *p* < 0.01. All models are adjusted for respondent age, education, number of children, relationship duration, partner’s age, partner’s education, partner controlling behaviour, and partner substance use.

## Data Availability

The datasets used for this research are accessible at https://dhsprogram.com/data/available-datasets.cfm (accessed on 31 August 2024).
